# Combining Machine Learning, Patient-Reported Outcomes, and Value-Based Health Care: Protocol for Scoping Reviews

**DOI:** 10.2196/36395

**Published:** 2022-07-18

**Authors:** Tyler Raclin, Amy Price, Christopher Stave, Eugenia Lee, Biren Reddy, Junsung Kim, Larry Chu

**Affiliations:** 1 Department of Anaesthesiology Stanford School of Medicine Stanford, CA United States; 2 Lane Medical Library Department Stanford University Stanford, CA United States; 3 University of Chicago Chicago, IL United States

**Keywords:** machine learning, artificial intelligence, algorithm, eHealth, patient-reported outcome measures, patient-reported experience measures, patient experience, value-based care, scoping review

## Abstract

**Background:**

Patient-reported outcome measures (PROMs) and patient-reported experience measures (PREMs) are self-reporting tools that can measure important information about patients, such as health priorities, experience, and perception of outcome. The use of traditional objective measures such as vital signs and lab values can be supplemented with these self-reported patient measures to provide a more complete picture of a patient’s health status. Machine learning, the use of computer algorithms that improve automatically through experience, is a powerful tool in health care that often does not use subjective information shared by patients. However, machine learning has largely been based on objective measures and has been developed without patient or public input. Algorithms often do not have access to critical information from patients and may be missing priorities and measures that matter to patients. Combining objective measures with patient-reported measures can improve the ability of machine learning algorithms to assess patients’ health status and improve the delivery of health care.

**Objective:**

The objective of this scoping review is to identify gaps and benefits in the way machine learning is integrated with patient-reported outcomes for the development of improved public and patient partnerships in research and health care.

**Methods:**

We reviewed the following 3 questions to learn from existing literature about the reported gaps and best methods for combining machine learning and patient-reported outcomes: (1) How are the public engaged as involved partners in the development of artificial intelligence in medicine? (2) What examples of good practice can we identify for the integration of PROMs into machine learning algorithms? (3) How has value-based health care influenced the development of artificial intelligence in health care? We searched Ovid MEDLINE(R), Embase, PsycINFO, Science Citation Index, Cochrane Library, and Database of Abstracts of Reviews of Effects in addition to PROSPERO and the ClinicalTrials website. The authors will use Covidence to screen titles and abstracts and to conduct the review. We will include systematic reviews and overviews published in any language and may explore additional study types. Quantitative, qualitative, and mixed methods studies are included in the reviews.

**Results:**

The search is completed, and Covidence software will be used to work collaboratively. We will report the review using the PRISMA (Preferred Reporting Items for Systematic Reviews and Meta-Analyses) guidelines and Critical Appraisal Skills Programme for systematic reviews.

**Conclusions:**

Findings from our review will help us identify examples of good practice for how to involve the public in the development of machine learning systems as well as interventions and outcomes that have used PROMs and PREMs.

**International Registered Report Identifier (IRRID):**

DERR1-10.2196/36395

## Introduction

Objective measures such as vital signs and lab values only provide a partial view of a patient’s condition. Patient-reported outcome measures (PROMs) [1] and patient-reported experience measures (PREMs) [1] are subjective reports shared by patients that can help complete this view by filling in gaps that other methods are incapable of assessing, such as pain levels, patient experience, motivation, human factors, patient-related outcomes, and health priorities. PROMs are questionnaires measuring the patients’ views of their health status. PREMs refer to data collected from patients about their experience within the health care system. These questionnaires can help us understand the patient perspective to identify goals for care and evaluate the impact of care.

Furthermore, earlier implementations of machine learning in medicine were developed without patient or public input and may be missing priorities and measures that matter to patients. Public and patient involvement can bring these measures together by defining end-user experience, meaning patient priorities implementation, and therefore provide enriched data for machine learning and more functional PROMs and PREMs.

Machine learning is an application of artificial intelligence (AI) that trains systems to automatically learn and improve from experience. In the past decade, machine learning has given us practical speech and speech-to-text recognition, algorithms for medical diagnosis, improvements in predictive epidemiology and public health, and prognostic treatment models. Although this is a powerful tool, these algorithms are only as reliable and free from bias as the data that are used to build and train them [2].

This review of reviews looks at ways to integrate machine learning with patient-reported outcomes for the development of improved public and patient partnerships in research and health care.

## Methods

### Review Questions

In this review, we will address the following 3 specific questions to learn about the best methods for combining machine learning and patient-reported outcomes:

- How are the public engaged as involved partners in the development of AI in medicine?
- What examples of good practice can we identify for the integration of PROMs into machine learning algorithms?
- How has value-based health care influenced the development of AI in health care?

### Searches

This review covers a broad range of interrelated topics, and we will assess the overall data by conducting 3 separate scoping reviews. The first review will focus on the intersection of AI and PROMs. The second scoping review will focus on AI and public involvement. The third one will focus on AI and value-based health care. We have chosen to do 3 separate scoping reviews instead of 1 or multiple systematic reviews to more efficiently identify knowledge gaps and investigate the way the research was conducted [3,4]. Preliminary searches have indicated that large bodies of knowledge have been published concerning the integration of PROMs into statistical methods [5-7], but few have indicated frameworks for public and patient involvement in the development of AI. Search strategies for each review were developed by the team and reviewed by our information specialist (CS). Our search strategies use controlled terms and a range of techniques to optimize sensitivity. No language restrictions will be applied. Each review will include relevant date restrictions to further isolate informative and innovative research.

The MEDLINE database will be used to identify initial search results. Initial search results will be reviewed to confirm there are no significant exclusions. Once the final search strategy has been identified, we will expand our search to the following information sources: Ovid MEDLINE(R), Embase, PsycINFO, Science Citation Index, Cochrane Library, Database of Abstracts of Reviews of Effects, and PROSPERO (International Prospective Register of Systematic Reviews). Search strategies can be viewed in Multimedia Appendix 1.

### Inclusion Criteria

We will include systematic reviews and overviews published in any language. Reviews will be included if they have searched a minimum of 2 databases, appraised the included studies, and provided a synthesis of the data and information retrieved. All findings will be reviewed and discussed by members of the author team until a consensus is reached. Once a preliminary set of eligible studies has been identified for each review based on outcome measures and broad inclusion criteria, we will progress to the next stage of evaluation. Each eligible study will be further evaluated based on narrower inclusion criteria to select the most relevant and informative research for each review. Narrower inclusion criteria will be specific to each research question. For the public engagement question, articles will be eligible if they discuss the involvement of the public in the development of AI or machine learning in medicine. For the question about examples of good practice, articles will be eligible if they discuss examples of integration of PROMs into machine learning algorithms. For the value-based care question, articles will be eligible if they discuss how value-based health care has or will impact the development of AI.

### Exclusion Criteria

Upon initial screening of titles and abstracts, we will exclude articles meeting any of the following criteria:

- Papers not dealing with any form of or related forms of AI
- Papers in which no relevant outcomes are reported
- Papers describing protocols for future studies
- Papers dealing with animal models
- Papers for which the full text is not accessible
- Papers that are not directly related to health care
- Papers that are theoretical models not tested on people
- Papers that are only about the methods of AI

### Condition or Domain Being Studied

We are investigating 3 domains. In the first scoping review, we will study examples of how the general public has been involved in AI development, where the outcomes include aspects of the trial and the experiences and perspectives of the public, participants, or researchers. The second review will focus on machine learning algorithms that have used PROMs to improve their performance in a health care–related task. This will include any research that is investigating the use of PROMs to improve diagnostic or treatment approaches. We will include an analysis of the time and length of each study, and whether the research included a plan for protecting patient-generated data. The third review will investigate AI research that has focused on value-based care. Studies that have used AI to investigate, evaluate, or design value-based care systems will be included.

### Data Extraction

The flow of information through different stages of our review will be guided by the PRISMA (Preferred Reporting Items for Systematic Reviews and Meta-Analyses) flowchart [8]. First, we will identify records through database searching and other sources, as described in Multimedia Appendix 1. Relevant results from each database and source of information will then be downloaded into Zotero (version 5.0.96.2; Corporation for Digital Scholarship), a management software for managing research materials. Results will then be uploaded into Covidence (Veritas Health Innovation), a web-based tool for screening references, for screening and analysis. After uploading them into Covidence, we will remove duplicate records. Titles and abstracts of potentially relevant articles will then be screened independently by at least 2 reviewers against the relevant inclusion criteria. Discrepancies will be resolved through discussion with the entire group, when necessary. Individuals recruited from the Cochrane Task Exchange, Stanford Medicine X, and Stanford Science Technology and Medicine Summer Internship will coproduce the study design and will be active in screening, data extraction, analysis, and prioritizing what to report, as well as editing and authoring tasks.

After excluding initial search results that do not meet our inclusion criteria, we will begin to review the full text of included records. Full-text review will be conducted by at least 2 authors, with an additional author reserved to mediate areas where agreement is uncertain. The authors will then come to an agreement through discussion. The full-paper review will result in the final set of included records. The authors will provide tables to show the characteristics of the included studies, similar to Table 1, and an additional table to show the author, year, and exclusion reason for excluded full studies, similar to Table 2.

**Table 1:** Table showing selected characteristics identified in included studies.

Study name	Characteristics					
	Intervention	Enablers	Barriers	Outcomes	Results	Time and length of study
Example study	Intervention used	Enabler to intervention, if any	Barrier to intervention, if any	Measures of outcome used	Result of intervention	Time and length of intervention

**Table 2:** Table of excluded studies.

Study name and year	Exclusion reason
Example study	Reason for exclusion

### Public and Patient Involvement

Patients and members of the public will be involved in the review and will be trained to screen titles and abstracts, as well as conduct the risk of bias assessment. They will be mentioned as coauthors if they have met the standards for authorship. If they do not fulfill authorship criteria, they will be mentioned and thanked in the acknowledgments. Funding constraints and COVID-19 restrictions prevented us from involving them more actively in protocol building.

### Dissemination

The research will be disseminated via social media and presented by the authors at conferences and convenings. The lessons learned and the findings will be used to teach our teenage and young adult learners at Stanford Anesthesia Summer Institute.

### Main Outcomes

The following outcomes will be considered:

- Public involvement in AI research planning, conduct, or management
- Public involvement in research analysis
- Research recruitment, enrollment, and retention
- Factors that affect cooperation and participation
- PROMs
- PREMs
- Ethics related to the inclusion of patient-reported information in AI
- Factors relating to participant interaction with AI
- Barriers to acquiring PROMs and PREMs for use in AI research
- Cost-effectiveness outcomes relating to the inclusion of PROMs and PREMs in AI research

### Measures of Effect

Quantitative, qualitative, and mixed methods studies will be included in our reviews. If sufficient quantitative studies relating to the inclusion of PROMs in AI warrant a meta-analysis, we will perform it and calculate a weighted effect across the studies, using a random effects model. Depending on the type of patient-reported data collected, it may be useful to pool the data using an inverse variance method and analyze it using a random effects model. This may allow us to calculate statistical measures on PROMs, such as patients’ symptoms, patient function, and physician communication [9]. After using a random effects model, it may still be desirable to identify sources of heterogeneity. If this is the case, we will use a subgroup analysis approach to investigate the reasons for heterogeneity. In the event of high heterogeneity, which is common in an emergent field, we will report data descriptively and include the insights found from the included mixed methods and qualitative narrative review papers.

### Risk of Bias (Quality) Assessment

For quantitative studies, we will use the Grading of Recommendations, Assessment, Development, and Evaluation (GRADE) approach [10]. This approach provides a structured and transparent evaluation for summarizing the evidence for reviews. The GRADE approach classifies the quality of evidence of quantitative studies into one of 4 levels of high, moderate, low, and very low. The ratings of the quality of evidence describe how much confidence there is that the true effect lies close to that of the estimated effect.

Confidence in the Evidence from Reviews of Qualitative Research (CERQual) [11,12] will be used to summarize confidence in the findings of the qualitative reviews. This is based on the following 4 components: limitations of the methodology, relevance to the research question, coherence, and the adequacy of the data presented. CERQual enables ratings of “high,” “medium,” “low,” and “very low”. The starting point of “high confidence” reflects that each review finding is a reasonable representation of the question of interest, and it is downgraded if there are factors that would weaken this assumption. After assessing all 4 components independently, 2 authors will agree on the overall confidence for each review finding and the relevance to the review of reviews.

### Strategy for Data Synthesis

**Figure 1:**
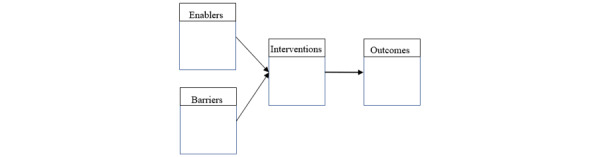
Flow chart indicating how enablers and barriers contribute to interventions and outcomes.

For the study investigating public involvement, we will use a relational analysis to present our results. Broadly, a relational analysis is a type of content analysis in which concepts found in our review will be further analyzed by how they relate to each other. This may show us how data are managed or protected and who has access to the data. We are most interested in approaches to public involvement in AI research, as well as enablers and barriers to those approaches. With this technique, we will be able to use data from eligible sources to identify examples of strategies, enablers, barriers, and outcomes. Once we have identified these examples in our eligible sources of information, we will be able to visually present these data in a flowchart and discuss these observations within the discussion section. The template for how this chart will look can be seen in Figure 1.

For the review focusing on PROMs, we will chart the difference between the evidence used and the outcomes collected for different algorithms that use PROMs, as seen in Table 3. In this review, we are most interested in how PROMs are integrated into AI tools and what outcomes result from their use. Finally, for the study focusing on value-based care, we will use a table similar to Table 3.

**Table 3:** Evidence and outcomes collected from included studies.

Reference	Evidence used				Outcomes collected		
	Evidence 1	Evidence 2	Evidence 3	Outcome 1		Outcome 2	Outcome 3
Example study 1	✓		✓	✓			✓
Example study 2		✓				✓	

### Ethics Approval

Our unfunded scoping review was exempted from Stanford Institutional Review Board approval, as it does not access personal health information, and data are synthesized from already published materials.

## Results

By executing this proposed protocol, we are hoping to identify examples of good practice for how to include public involvement in the development of machine learning systems. We hope to identify enablers and barriers to public involvement, as well as interventions and outcomes that have used PROMs and PREMs. Lastly, we hope to identify examples of how value-based health care has influenced the development of AI systems in health care.

## Discussion

### Limitations

We have chosen to do 3 scoping reviews instead of full systematic reviews because research has indicated that scoping reviews will help us answer our research questions more efficiently [3]. In a scoping review, the goal is to determine what evidence is available rather than synthesizing evidence from multiple study designs and providing concrete guidance [13]. This is because scoping reviews are limited in their ability to provide concrete guidance. However, we are only aiming to examine the types of available evidence in this field and identify the key factors related to our topics. We are attempting to identify methods to include patient involvement in machine learning and explore how value-based care has impacted machine learning. We are not aiming to produce a specific answer to a specific clinical or policy-making question. Therefore, this limitation is acceptable.

Furthermore, scoping reviews generally provide an overview of existing evidence regardless of quality [13]. In our scoping reviews, we will be using the GRADE approach and CERQual to assess the quality of our sources. In this approach, we will discuss the qualities of reviews and determine whether the review should be included. Thus, we are directly addressing this limitation and still believe a scoping review is the right choice for each topic.

### Conclusions

This protocol outlines our methods for 3 scoping reviews of published literature to discover effective strategies for the development of improved public and patient partnerships in AI and machine learning in research and health care.

## Appendix

Multimedia Appendix 1 Search strategies.

## Abbreviations

AI: artificial intelligence
CERQual: Confidence in the Evidence from Reviews of Qualitative Research
GRADE: Grading of Recommendations Assessment, Development, and Evaluation
PREM: patient-reported experience measure
PRISMA: Preferred Reporting Items for Systematic Reviews and Meta-Analyses
PROM: patient-reported outcome measure
PROSPERO: International Prospective Register of Systematic Reviews
