# Supplementary material for: Combining Machine Learning, Patient-Reported Outcomes, and Value-Based Health Care: Protocol for Scoping Reviews
Source: JMIR Res Protoc. 2022 Jul 18;11(7):e36395. doi: 10.2196/36395 (PMC9345029; doi:10.2196/36395)
Supplement: Multimedia Appendix 1 [file resprot_v11i7e36395_app1.docx]

Appendix 1: Search Strategies

We searched Ovid MEDLINE(R), EMBASE, PsycINFO, Science Citation Index, Cochrane Library, Database of Abstracts of Reviews of Effects in addition to PROSPERO, and ClinicalTrials.gov. The patient reported outcome string refers to searches conducted for our first research question, public involvement for the second, and value based health care for the third.

These would all be AND our AI string e.g.

1. AI string and Patient Reported Outcome
2. AI string and Public Involvement
3. Ai string and Value Based Care

**PUBMED:**

**AI**

exp artificial Intelligence/ OR exp big data/ OR exp machine learning/ OR exp data mining/ OR exp Neural Networks, Computer/ OR exp deep learning/ OR "artificial intelligence".mp. or "neural network".mp. or "convolutional neural network".mp. or "deep learning".mp. or "prediction model*".mp or "predictive model*".mp or "machine learning".mp. or "iterative methods".mp. or "iterative reconstruction".mp. or "data mining".mp OR "adversarial network*".mp

**Patient Reported Outcomes**

exp patient outcome assessment OR (Patient reported outcome* or person reported outcome* or patient reported experience* or person reported experience* or PRO or PROM or PROMIS or PREM).mp OR ((patient satisfaction or person* satisfaction) adj (assess* or scale or questionnaire* or screen* or inventor* or index or indices or instrument* or inventor* or measure* or tool*)).mp. OR ((self-report* or self assess*) adj3 (quality of life or QoL or HRQoL or HR QoL or HQoL)).mp

LEAVE OFF* (regression model* or model fit or analytical model* or random effects model* or adjusted model* or coefficent model* or co-efficient model* or joint model* or predictive model* or (linear adj2 model*) or (rasch adj3 model*) or (cox adj3 model*)).mp.

**Public Involvement**

exp patient participation/ OR ((public or patient* or citizen* or survivor* or volunteer* or consumer* or user* or stakeholder*) adj (involv* or participat* or engag* or collaborat* or cooperat* or co-operat* or research)).mp. OR (expert adj (patient* or user* or consumer*)).mp. OR ((public or patient* or citizen* or survivor* or volunteer* or consumer* or user* or stakeholder*) adj (panel* or group*)).mp.

**Value Based Health Care**

("value based care" OR "value based healthcare" OR "value based health" OR "value based perform*" OR "value driven care" OR "value driven healthcare" OR "value driven health" OR "value driven perform*" ).mp OR exp value based health Insurance

**EMBASE:**

**AI**

('artificial intelligence'/exp OR 'data mining'/exp OR 'big data'/exp OR 'machine learning'/exp OR 'neural networks'/exp OR 'deep learning'/exp OR 'neural networks'/exp OR 'prediction model'/exp OR 'artificial neural network'/exp OR "artificial intelligence":ti,ab,kw OR "neural network":ti,ab,kw OR "convolutional neural network":ti,ab,kw OR "deep learning":ti,ab,kw OR "prediction model*":ti,ab,kw OR "predictive model*":ti,ab,kw OR "machine learning":ti,ab,kw OR "iterative methods":ti,ab,kw OR "iterative reconstruction":ti,ab,kw OR "data mining":ti,ab,kw OR "adversarial network*":ti,ab,kw) AND [embase]/lim NOT ([embase]/lim AND [medline]/lim) AND [english]/lim

**Patient Reported Outcomes**

(('patient-reported outcome'/exp OR "Patient reported outcome*":ti,ab,kw OR "person reported outcome*":ti,ab,kw OR "patient reported experience*":ti,ab,kw OR "person reported experience*":ti,ab,kw OR PRO:ti,kw OR PROM:ti,kw OR PROMIS:ti,kw OR PREM:ti,kw)) OR (("patient satisfaction" OR "personal satisfaction") NEAR/1 (assess* OR scale OR questionnaire* OR screen* OR inventor* OR index OR indices OR instrument* OR measure* OR tool*)) OR (("self-report*" OR "self assess*") NEAR/3 ("quality of life" OR QoL OR HRQoL OR "HR qol")) AND [embase]/lim NOT ([embase]/lim AND [medline]/lim) AND [english]/lim

**Public Involvement**

('patient participation'/exp) OR ((public or patient* or citizen* or survivor* or volunteer* or consumer* or user* or stakeholder*) NEAR/1 (involv* or participat* or engag* or collaborat* or cooperat* or co-operat* or research)) OR (expert NEAR/1 (patient* or user* or consumer*)) OR

((public or patient* or citizen* or survivor* or volunteer* or consumer* or user* or stakeholder*) NEAR/1 (panel* or group*)) AND [embase]/lim NOT ([embase]/lim AND [medline]/lim) AND [english]/lim

**Value Based Health Care**

'value based health care'/exp OR ("value based care":ti,ab,kw OR "value based healthcare":ti,ab,kw OR "value based health":ti,ab,kw OR "value based perform*":ti,ab,kw OR "value driven care":ti,ab,kw OR "value driven healthcare":ti,ab,kw OR "value driven health":ti,ab,kw OR "value driven perform*":ti,ab,kw) AND [embase]/lim NOT ([embase]/lim AND [medline]/lim) AND [english]/lim

**WEB OF SCIENCE**

**Public Involvement** USE TITLE SEARCH

("artificial intelligence" OR "neural network" OR "convolutional neural network" OR "deep learning" OR "prediction model*" OR "predictive model*" OR "machine learning" OR "iterative methods" OR "iterative reconstruction" OR "data mining" OR "adversarial network*") AND ((((public or patient* or citizen* or survivor* or volunteer* or consumer* or stakeholder*) NEAR/1 (involv* or participat* or engag* or collaborat* or cooperat* or co-operat* or research))) OR ((expert NEAR/1 (patient* or consumer*)) OR ((public or patient* or citizen* or survivor* or volunteer* or consumer* or user* or stakeholder*) NEAR/1 (panel* or group*))))

**AI + Patient Reported Outcomes** USE TITLE SEARCH

("artificial intelligence" OR "neural network" OR "convolutional neural network" OR "deep learning" OR "prediction model*" OR "predictive model*" OR "machine learning" OR "iterative methods" OR "iterative reconstruction" OR "data mining" OR "adversarial network*") AND (("Patient reported outcome*" OR "person reported outcome*" OR "patient reported experience*" OR "person reported experience*" OR PRO OR PROM OR PROMIS OR PREM) OR (("patient satisfaction" OR "personal satisfaction") NEAR/1 (assess* OR scale OR questionnaire* OR screen* OR inventor* OR index OR indices OR instrument* OR measure* OR tool*)))

**Value Based Health Care** USE TOPIC SEARCH:

("artificial intelligence" OR "neural network" OR "convolutional neural network" OR "deep learning" OR "prediction model*" OR "predictive model*" OR "machine learning" OR "iterative methods" OR "iterative reconstruction" OR "data mining" OR "adversarial network*") AND ("value based care" OR "value based healthcare" OR "value based health" OR "value based perform*" OR "value driven care" OR "value driven healthcare" OR "value driven health" OR "value driven perform*")

**COCHRANE LIBRARY**

**Value based Care**

("artificial intelligence" OR "neural network" OR "convolutional neural network" OR "deep learning" OR "prediction model*" OR "predictive model*" OR "machine learning" OR "iterative methods" OR "iterative reconstruction" OR "data mining" OR "adversarial network*") AND ("value based care" OR "value based healthcare" OR "value based health" OR "value based perform*" OR "value driven care" OR "value driven healthcare" OR "value driven health" OR "value driven perform*")

**AI and PRCs**

("artificial intelligence" OR "neural network" OR "convolutional neural network" OR "deep learning" OR "prediction model*" OR "predictive model*" OR "machine learning" OR "iterative methods" OR "iterative reconstruction" OR "data mining" OR "adversarial network*") AND ("Patient reported outcome*" OR "person reported outcome*" OR "patient reported experience*" OR "person reported experience*" OR PRO OR PROM OR PROMIS OR PREMOR "patient satisfaction" OR "personal satisfaction")

**AI and Patient Involvement:**

("artificial intelligence" OR "neural network" OR "convolutional neural network" OR "deep learning" OR "prediction model*" OR "predictive model*" OR "machine learning" OR "iterative methods" OR "iterative reconstruction" OR "data mining" OR "adversarial network*") AND ("patient Involvement" OR "patient participation" OR "patient engagement" OR stakeholder OR consumer)

**CINAHL**

**AI**

(TI ("artificial intelligence" OR "neural network" OR "convolutional neural network" OR "deep learning" OR "prediction model*" OR "predictive model*" OR "machine learning" OR "iterative methods" OR "iterative reconstruction" OR "data mining" OR "adversarial network*") OR MH ("Artificial Intelligence+" OR "data mining+" OR "deep learning+" OR "machine learning+:)) AND LA (english)

**PRC**

(TI ("Patient reported outcome*" OR "person reported outcome*" OR "patient reported experience*" OR "person reported experience*" OR PRO OR PROM OR PROMIS OR PREMOR "patient satisfaction" OR "personal satisfaction") OR MH ("Patient-Reported Outcomes+")) AND LA (english)

**Patient Involvement**

TI ("patient Involvement" OR "patient participation" OR "patient engagement" OR stakeholder OR consumer) OR MH ("consumer participation+") AND LA (english)
